# UPLC-TOF/MS-based metabolomics reveals the chemical changes and *in vitro* biological effects in fermentation of white ginseng by four probiotics

**DOI:** 10.3389/fmicb.2022.1022200

**Published:** 2022-11-24

**Authors:** Yincui Chen, Yunsheng Wang, Anqin Zhu, Liqin Zhang, Xiang Zhang, Jin Zhang, Chuanbo Zhang

**Affiliations:** Laboratory of Microbial Resources and Industrial Application, College of Life Sciences, Guizhou Normal University, Guiyang, China

**Keywords:** white ginseng, microbial fermentation, antioxidant activity, hypoglycemic activity, metabolomics

## Abstract

Microbial fermentation is a useful method for improving the biological activity of Chinese herbal medicine. Herein, we revealed the effects of solid-state fermentation by *Lactiplantibacillus plantarum*, *Bacillus licheniformis*, *Saccharomyces cerevisiae*, *Eurotium cristatum* and multiple strains on total flavonoid content, total phenol content, as well as antioxidants, *α*-amylase inhibitory activities and *α*-glucosidase inhibitory activities in white ginseng (WG). Metabolite differences between non-fermented and fermented WG by different probiotics were comprehensively investigated using ultra-performance liquid chromatography time-of-flight mass spectrometry (UPLC-TOF-MS). Results showed that the total flavonoid content, ferric reducing antioxidant power, scavenging activities of DPPH radical and ABTS radical, *α*-amylase inhibitory activities and *α*-glucosidase inhibitory activities of WG were considerably enhanced after processing by solid-state fermentation in all strains. The total phenol content was increased by *E. cristatum* and *B. licheniformis* fermentation, but decreased by *L. plantarum*, *S. cerevisiae* and multi-strain fermentation. Additionally, *E. cristatum* exhibited stronger biotransformation activity on WG compared to other strains. Significant differential metabolites were mainly annotated as prenol lipids, carboxylic acids and derivatives, flavonoids, polyphenols, coumarins and derivatives. Correlation analysis further showed that changes of these metabolites were closely related to antioxidant and hypoglycemic effects. Our results confirmed that fermentation of WG by different probiotics has distinct effects on biological activities and metabolite composition, and indicating fermentation as an important novel strategy to promote components and bioactivities of WG.

## Introduction

The ginseng root of *Panax ginseng* C.A. Meyer from a perennial plant, one of the most traditional medicines in East Asia, was widely used as an ingredient for functional foods and health promoting owing to its various active components (Dong et al., 2019; Yoon et al., 2020; Jin et al., 2021). According to the different manufacturing processes, ginseng can be divided into four types: white ginseng (WG), tae-geuk ginseng (TG), red ginseng (RG) and black ginseng (BG) (Zhang et al., 2012; Lee et al., 2018; Yoon et al., 2020). Saponin, flavonoid and polysaccharide content in different types of ginseng varied with the distinct processing methods. Traditionally, WG is produced by dehydrating raw ginseng in sunlight, which is considered to be a relatively mild method and has the specific function of quenching thirst (Pang et al., 2016; He et al., 2018). The main biological activity components of ginseng are saponins, flavonoids, amino acids and other nutrients. Interestingly, previous study revealed that the content of total saponins and monomer saponins Rg1, Re, Rb1and Rc in WG were significantly higher than RG and BG, and the total flavone content in WG was higher than RG (Shibata, 2001; Wu et al., 2015). Furthermore, the water extract of WG contains carbohydrate with specific structure and composition, and its polysaccharide was mainly starch-like glucans, which had a strong anti-obesity effect on obese mice (Zhou et al., 2020). Altogether, these results indicate that WG is a desirable source of medicinal substances.

Saponins are the main characteristic components of ginseng, which are condensates of isoprene units with isoprenol as the mother nucleus (Fahy et al., 2005). The main ginsenosides in ginseng, such as Rb1, Rb2, Rd., Re and Rg1, account for more than 80%, which have low pharmacological activity and little absorption in the human body (Kim et al., 2012). Recently, many researchers have focused on the use of microorganisms and enzymes to strengthen biological activities and the utilization rate of effective ingredients of ginseng (e.g., anti-fatigue, anti-oxidation, anti-aging, anti-allergy, anti-diabetes and improving immunity) by converting ginsenosides into smaller deglycosylated forms, generating rare ginsenosides, and increasing the content of active substances (Yu et al., 2017; Wang et al., 2020). Ginsenoside Rb1 could be hydrolyzed to minor ginsenosides Rd. and Rg3 by fermentation with *Lentilactobacillus buchneri* (Renchinkhand et al., 2022). In addition, the fermentation of red ginseng with *Lactiplantibacillus plantarum* could transform ginsenoside Rb2 and Rb3 into Rd., which enhanced the antioxidant activity of RG (Jung et al., 2017). Furthermore, fermentation with *Saccharomyces cerevisiae* could dramatically increase the antioxidant activity of BG compared with non-fermentation group (Jung et al., 2019).

As well-known probiotics, *L. plantarum* and *Bacillus licheniformis* are widely used as a starter culture in various food fermentation processes, such as yogurt, pickled vegetables and Chinese liquor making, contributing to improve food quality and sensory properties (Behera et al., 2018; Wang et al., 2021; Chen and Yu, 2022). *L. plantarum* produces abundant enzymes, such as *β*-glucosidase, amylase and proteinase, which aided the increase of phenol content in smoothies and soy after fermentation (Lee and Bae, 2019; Managa et al., 2021). Similarly, *B*. *licheniformis* fermentation significantly increased phenol and pyrazine content in soybean meal, owing to its extracellular enzyme activity (Dai et al., 2022). These results suggest that fermentation by *L. plantarum* and *B. licheniformis* is an effective process to improve nourishing and functional food ingredients. *S. cerevisiae*, is one of the most important microbial species with superior fermentation performance, which is a prerequisite across a range of industries such as baking, brewing, traditional fermented food (Eldarov et al., 2016). *Eurotium cristatum* has been reported to play an important role in the “flowering” process of Fu brick tea, its rich enzymes exhibit a strong biological transformation ability on a variety of plant substrates, such as tea, soybean dregs, ginkgo seeds, etc. (Chen et al., 2020; An et al., 2021; Zou et al., 2021).

In present work, the four probiotics (*E. cristatum*, *L. plantarum*, *B. licheniformis* and *S. cerevisiae*) and a mixture of these strains were selected to ferment WG. The effect of the total phenol content, flavonoids content, antioxidant potential and hypoglycemic activity of fermented WG with these four strains compared to unfermented WG were evaluated. We used an untargeted metabolomics approach to further analyze changes in metabolite profiles in fermented WG with different strains, and elaborate the correlation between metabolites and biological activities. The purpose of this study was to establish an effective processing technology for strengthening the biological activity of WG, and provide novel insights into the microbial metabolism and bioconversion of ginseng products.

## Materials and methods

### Chemicals and reagents

Gallic acid, 2,2-azinobis(3-ethylbenzothiazoline-6-sulphonicacid) (ABTS), 2,2-diphenyl-1-picrylhydrazyl (DPPH), potassium peroxodisulfate, ferrous sulphate hepta-hydrate, 2,4,6-tripyridyl-s-triazine (TPTZ), *α*-amylase, *α*-glucosidase and *p*-nitrophenyl-β-D-glucoside (*ρ*NPG) were purchased from Solarbio Bioscience & Technology Co., Ltd. (Shanghai, China). All other chemicals and reagents (e.g., acarbose, disodium phosphate, potassium dihydrogen phosphate, sodium carbonate, biodegradable organic carbon, a sole iron precursor, soluble starch, hydrogen chloride, ethanol, methanol, caustic soda, 3,5-dinitrosalicylic acid, potassium sodium tartrate, phenol and sodium sulphite) used in this study were analytical grade. Fresh ginseng was purchased from a supermarket in Tonghua city, Jilin Province, China.

### Strains and cultivation

The fungus *Eurotium cristatum*, previously isolated from Anhua dark tea in our laboratory, was cultivated on potato glucose agar medium (PDA, potato powder 0.6% (w/v), glucose 2% (w/v), and agar 2% (w/v)). *Bacillus licheniformis* and *Saccharomyces cerevisiae* were isolated and purified from fermented grains of Guizhou Guijiu Co., Ltd. (Guiyang, China), which were cultivated on Lennox broth liquid medium (LB, peptone 1% (w/v), NaCl 1% (w/v) and yeast extract 0.5% (w/v)) and PDA medium, respectively. *Lactiplantibacillus plantarum* was isolated and purified from the sour soup (Kaili, Guizhou) and cultivated on Man Rogosa Sharpe medium (MRS, peptone 1% (w/v), beef powder 0.8% (w/v), yeast powder 0.4% (w/v), glucose 2% (w/v), dimethyl hydrogen phosphate 0.2% (w/v), diammonium hydrogen citrate 0.2% (w/v), sodium acetate 0.5% (w/v), magnesium sulfate 0.05% g (w/v), manganese sulfate 0.004% (w/v), tween 80 1.5% (v/v) and agar 1.5% (w/v)).

*E. cristatum* and *S. cerevisiae* were inoculated in PDA medium, cultured at 30°C for 6 d and 24 h, respectively. The bacteria *L. plantarum* and *B. licheniformis* were inoculated in MRS solid medium and LB solid medium, followed by incubation at 37°C for 18 h and 30°C for 24 h, respectively. Before inoculation, microbial cells were washed thrice with sterile water, and suspensions were adjusted to 4 × 10^6^ spore/ml, 7.3 × 10^7^ CFU/ml, 1 × 10^7^ CFU/ml and 1 × 10^7^ CFU/ml by using a blood cell count board, respectively. The number of spores or cells of strains were obtained according to extensive preliminary experiments that focused on the estimation of strain growth characteristics and antioxidant activity of WG, which are not shown in this study.

### Solid-state fermentation of WG

WG was prepared by washing fresh ginseng, dehydrating in sunlight, and smashed with a grinder. Next, 20 g of WG powder was added to 300 ml Erlenmeyer flask and autoclaved at 115°C for 30 min. After cooling to room temperature, *E. cristatum*, *L. plantarum*, *B. licheniformis*, *S. cerevisiae* and multiple strains (5% v/w) were directly inoculated in WG for solid-state fermentation, and cultivated at 30°C for 6 d. Samples were referred to as EP, LP, BP, SP and MP, respectively. The uninoculated sterilized WG powder served as control, named PP.

### Solvent-extract preparation

WG products were dried in an air oven at 35°C for 3 d, crushed using a pulverizer and sieved through 10 mm mesh. Subsequently, 20 g of WG sample was added in 200 ml beaker, extracted thrice with 75% (v/v) ethanol by ultrasound (solid–liquid ratio is 1:8, soaked for 12 h), before being filtered and collected. Next, the sample was placed in a 35°C rotary evaporation instrument to obtain the extract, which was then stored in a 4°C refrigerator for subsequent analysis.

### Bioactivity assays

#### Total flavonoid content assay

The total flavonoid content of WG extracts was estimated by aluminum chloride method as described with a few modifications (Liu et al., 2020; Syed Salleh et al., 2021). The flavonoid content of WG extract was calculated using a rutin calibration curve (20, 10, 5, 2.5, 1.25, 0.625 and 0.3125 mg/ml). Briefly, 250 μl of sample was mixed with 125 μl NaNO_2_ (5%, w/v), the mixture was vortexed and left at room temperature for 6 min before the addition of 125 μl of Al (NO_3_)_3_ (10%, w/v), mixed for 6 min. Next, 1 ml NaOH (1%, w/v) was added to the solution and incubated for 15 min. The reaction was monitored by reading the absorbance at 510 nm. Total flavonoid content was expressed as rutin equivalents (mg/GAE g).

#### Total phenol content assay

The Folin–Ciocalteu method was used to determine the total phenol content in WG extracts as described with some modifications (Ainsworth and Gillespie, 2007). Briefly, 125 μl of sample was added to 50 μl Folin–Ciocalteu reagent and vortexed, before being incubated in the dark for 2 min. Subsequently, 500 μl sodium carbonate solution (10%, w/v) and 250 μl distilled water were added and mixed. Total phenol content was quantified at 750 nm reaction at 40°C for 30 min in a water bath in the dark. Total phenol content was expressed as gallic acid equivalents (mg/GAE g) referring to the gallic acid calibration curve (0.4, 0.2, 0.1, 0.05, 0.025, 0.0125 and 0.00625 mg/ml).

#### FRAP assay

A FRAP assay was performed according to Benzie and Lee with minor modifications (Benzie and Strain, 1996; Lee et al., 2021). The reducing capacity of WG extracts was calculated referring to the iron sulfate calibration curve (6.4, 3.2, 1.6, 0.8, 0.4 and 0.2 mg/ml) and expressed as the FRAP value (millimoles Fe (II) per gram sample). Simply, 5 μl of different concentrations of extract sample in 50% (v/v) methanol (4, 3, 2, 1, 0.5 and 0.25 mg/ml) were added to 180 μl acidic FRAP reagent (the reaction mixture was prepared by mixing 200 ml of 300 mM acetate buffer, pH 3.6, 20 ml of 10 mM TPTZ in 40 mM HCl, and 20 ml of 20 mM FeCl_3_). Next, the mixed solution was incubated at 37°C for 10 min, and the reaction was monitored by reading the absorbance at 593 nm.

#### ABTS radical scavenging activity assay

ABTS radical scavenging activity was monitored as previously described with slight modifications (Di Cagno et al., 2019; An et al., 2021). First, 7 mmol/l ABTS and 2.45 mmol/l K_2_S_2_O_8_ solution were mixed in equal volume (v:v) and incubated at room temperature for 16 h in the dark. Before use, the solution was diluted with anhydrous ethanol to reach an absorbance value of 0.70 (± 0.02) at 734 nm. Briefly, 50 μl of different concentrations of extract sample in 50% (v/v) methanol (5, 4, 3, 2, 1, 0.5 and 0.25 mg/ml) were mixed with 195 μl of ABTS solution. Samples were then kept in the dark for 6 min at room temperature before the reaction was monitored by microplate reader at 517 nm. The ABTS scavenging ability was calculated as a percentage as follows:


$$\begin{array}{l} \mathrm{ABTS}\;\;\mathrm{scavenging}\;\;\mathrm{ability}\;\left(\%\right)=\left(1-\frac{A_{1}-A_{2}}{A_{3}}\right)\times 100\% \end{array}$$

where A_1_ is the absorbance of the sample, A_2_ is the absorbance of the control, and A_3_ is the absorbance of the blank.

#### DPPH radical scavenging activity assay

The DPPH radical scavenging activity was measured as previously described with slight modifications (Zhang et al., 2014; Wu et al., 2017). Briefly, 100 μl of different concentrations of extract sample in 50% (v/v) methanol (6, 5, 4, 3, 2, 1 and 0.5 mg/ml) were mixed with 100 μl of 0.2 mM DPPH ethanolic solution. Samples were then incubated in the dark for 30 min at room temperature and the reaction was monitored by reading the absorbance at 517 nm. DPPH scavenging ability was calculated as a percentage as follows:


$$\begin{array}{l} \mathrm{DPPH}\;\;\mathrm{scavenging}\;\;\mathrm{ability}\;\;\left(\%\right)=\left(1-\frac{A_{1}-A_{2}}{A_{3}}\right)\times 100\% \end{array}$$

where A_1_ is the absorbance of the sample, A_2_ is the absorbance of the control, and A_3_ is the absorbance of the blank.

#### Inhibition assays of samples on *α*-amylase

The antidiabetic potential of WG extracts was evaluated using an *in vitro α*-amylase inhibitory assay as previously described with minor changes (Prasad et al., 2019). Simply, 100 μl of the sample extract in distilled water (1, 0.5, 0.25, 0.125, 0.0625, 0.03125 and 0.015625 mg/ml) was added to 100 μl of 1 unit/ml *α*-amylase. The mixture was then incubated at 37°C in a water bath for 10 min followed by addition of 100 μl of 1% starch (w/v) in distilled water before further incubation in a 37°C water bath for 10 min. Finally, 50 μl of 1% DNS was added and heated in a water bath for 5 min. The solution was cooled to room temperature and the sample absorbance was measured at 540 nm. Acarbose was used as positive control. The *α*-amylase inhibitory rate was calculated as a percentage as follows:


$$\begin{array}{l} \;\;\%\mathrm{inhibitory}\;\;=\left(1-\frac{A_{4}-A_{3}}{A_{2}-A_{1}}\right)\times 100\% \end{array}$$

where A_4_ is the absorbance of the sample, A_3_ is the absorbance of the experimental background, A_2_ is the absorbance of the control, and A_1_ is absorbance of the blank.

#### Inhibition assays of samples on *α*-glucosidase

The ability of WG extracts to inhibit *α*-glucosidase activity was assessed according to a previously described protocol with slight changes (Purnomo et al., 2021). Firstly, 20 μl the different sample extracts in distilled water (20, 16, 12, 8, 4, 2 and 1 mg/ml) were added to 100 μl of 0.1 unit/ml *α*-glucosidase and the mixture was incubated in a 37°C water bath for 10 min followed by addition of 50 μl of 5 mmol/l *ρ*NPG. The mixture was further incubated for 10 min at 37°C. Finally, the assay reaction was terminated by adding 0.1 M, 100 μl sodium carbonate. All samples were measured at 405 nm and corresponding absorbances were noted. Acarbose was used as a positive control. The *α*-glucosidase inhibitory rate was expressed as a percentage and calculated as follows:


$$\begin{array}{l} \%\;\;\mathrm{inhibitory}\;\;=\left(1-\frac{A_{4}-A_{3}}{A_{2}-A_{1}}\right)\times 100\% \end{array}$$

where A_4_ is the absorbance of the sample, A_3_ is the absorbance of the experimental background, A_2_ is the absorbance of the control, and A_1_ is the absorbance of the blank.

### Metabolomics analysis

The sample preparation process was the same as in Section 2.4. Sample metabolites were analyzed on a UHPLC-triple TOF system from B SCIEX. Compounds were separated on an ACQUITY UPLC HSS T3 column (100 mm × 2.1 mm, 1.8 μm) (Waters, Milford, USA). The sample injection volume was 10 μl, the column temperature was maintained at 40°C and the total separation time for chromatographic analysis was set as 16 min. Mobile phase A was 95% water +5% acetonitrile (0.1% formic acid in water) and mobile phase B was 47.5% acetonitrile +47.5% isopropanol +5% water (0.1% formic acid in water). The flow rate was set to 0.4 ml/min and the gradient program was as follows: 100% A (0–0.5 min), 75% A (0.5–2.5 min), 0% A (2.5–13 min), and 100% A (13–16 min). Sample mass spectrum signal acquisition was collected in positive and negative ion scanning modes. Optimum parameters for MS were set as follows: the mass range were set at m/z 50 ~ 1,000; the ion source gas1 and gas2 were both set at 50 psi with temperature of 550°C; the Ion Spray Voltage Floating was −4,000 V in negative mode and 5,000 V in positive mode. For metabolomic data analysis, the raw data were imported into the metabolomics software Progenesis QI (Waters Corporation, Milford, USA) for processing and identification of characteristic peaks according to the methods of Shi et al. (2020).

### Statistical analysis

All experiments were performed three times, and data were analyzed with GraphPad Prism 7 and IBM SPSS Statistics 23. For UPLC-TOF-MS analysis, principal component analysis (PCA) and partial least squares discriminant analysis (PLS-DA) were performed by using ropes (R packages) version1.6.2. Regarding results obtained using the PLS-DA model, the different metabolites between groups were identified according to the value (value >1) of variable importance in the projection (VIP), *t*-test analysis (*p <* 0.05) for the fold-change (FC) analysis (FC > 2 or < 0.5). Person correlation analysis between the functional characteristics and chemical constituents of WG samples were performed by ropls (R packages) and the heatmap was drawn using scipy (Python).

## Results

### Phenolic and flavonoid content

Here, the flavonoid content during the fermentation of WG by different probiotics was measured. The standard curve regression equations for rutin and gallic acid were y = 0.0515x + 0.0453 (R^2^ = 0.999 8), y = 9.1001x + 0.1698 (R^2^ = 0.999 0), respectively. Where y is absorbance and x is the rutin and gallic acid concentration in mg/ml. The phenolic and flavonoid content of EP, LP, BP, SP and MP are shown in Table 1. Compared to PP (1.17 ± 0.11 mg/g), the flavonoid content of EP (3.81 ± 0.27 mg/g), MP (2.84 ± 0.26 mg/g) and SP (2.04 ± 0.16 mg/g) were significantly increased (*p <* 0.05) by 3.26-, 2.43- and 1.74-fold, respectively. Moreover, total flavonoid content in the WG of LP and BP also showed an upward trend, but these were no significant differences.

**Table 1:** Effect of different fermentation group on the flavonoid and phenolic content.

Sample	Total flavonoids	Total phenolic content
	(mg of rutin/g of sample)	(mg of gallic acid/g of sample)
LP	1.69 ± 0.14	0.74 ± 0.05*
BP	1.30 ± 0.07	0.89 ± 0.02
SP	2.04 ± 0.16**	0.77 ± 0.03
EP	3.81 ± 0.27***	0.97 ± 0.02*
MP	2.84 ± 0.26***	0.78 ± 0.01
PP	1.17 ± 0.11	0.85 ± 0.01

Data were reported as the mean value ± standard deviation of three replicates. Different letters above the bars indicate significant differences (*p* < 0.05: *, *p* < 0.01: ** and *p* < 0.001: ***).

Compared to PP (0.85 ± 0.01 mg/g), the total phenol content of BP (0.89 ± 0.02 mg/g) was improved, but that in MP, SP and LP decreased. Interestingly, we found that the total phenol content of WG was significantly increased after *E. cristatum* fermentation (EP group, 0.97 ± 0.02 mg/g). These data showed that fermentation of *E. cristatum* increased total flavonoid and total phenol content in WG compared to *L. plantarum*, *B. licheniformis*, *S. cerevisiae* and the mixed group.

### Measurement antioxidant activities of WG extracts

**Figure 1:**
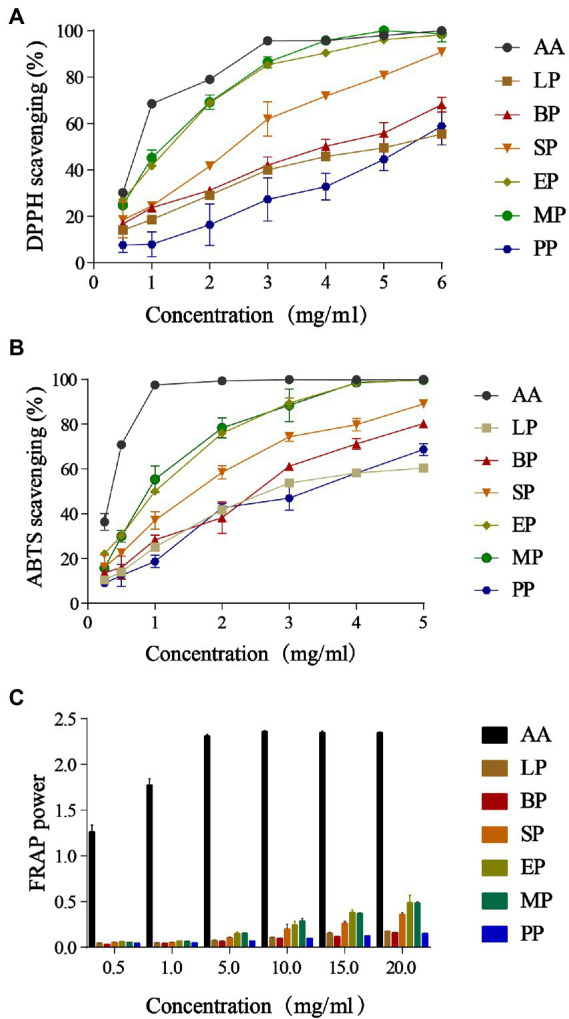
DPPH radicals scavenging activities **(A)**; ABTS radicals scavenging activities **(B)** and ferric reducing antioxidant power **(C)**. Data are reported as the mean value ± standard deviation of three replicates.

The DPPH method was used to evaluate the antioxidant activity of WG *in vitro*, the percentage of remaining DPPH against the extract concentration was plotted (Figure 1A). We found that the scavenging ability of DPPH radicals in WG extracts of the LP, SP, EP, MP and BP groups improved in a dose-dependent manner compared to the control (PP). The DPPH radical scavenging capacity of EP and MP was close to 1 mg/ml of ascorbic acid, and the DPPH radical scavenging rate was over 90% at 4 mg/ml of WG extracts. Importantly, when the concentration of the extract reached 6 mg/ml, the DPPH radical scavenging rate of EP and MP was close to 100%. These data showed that the DPPH radical scavenging ability of WG was improved by fermentation of *B. licheniformis, S. cerevisiae, E. cristatum, L. plantarum* and mixed probiotics. Remarkably, fermentation of *E. cristatum* and mixed group showed a significantly improved DPPH radical scavenging rate within the measured concentration range.

The effect of ABTS radical scavenging activity of different groups *in vitro* was shown in Figure 1B. We found that as the sample concentration increased ABTS free radical scavenging ability also increased in a dose-dependent manner. Compared to PP, both microbial fermentation groups (BP, SP, EP, LP and MP) improved the ABTS free radical scavenging ability of WG. Interestingly, EP and MP groups showed increased ABTS radical scavenging compared to other groups, with a scavenging rate close to 100% at 4 mg/ml.

The FRAP method was used in this work to evaluate the effect of microbial fermentation on the reducing power of the extracts of WG. As shown in Figure 1C, the total reducing power of LP, SP, EP and MP groups were significantly increased (*p* < 0.05) at different concentrations compared to the control group. Consistent with the above results, total reducing power increased in the EP and MP groups compared to the other groups, with absorbance at 0.5–20 mg/ml ranging from 0.062–0.488 and 0.056–0.485, respectively. These data indicated that microbial fermentation was an ideal method to improve the antioxidant activity of WG products. Moreover, within the measured concentration range, *E. cristatum* and multi-strain fermentation performed better.

### Inhibitory effect of extracts on the activity of *α*-amylase and *α*-glucosidase

**Figure 2:**
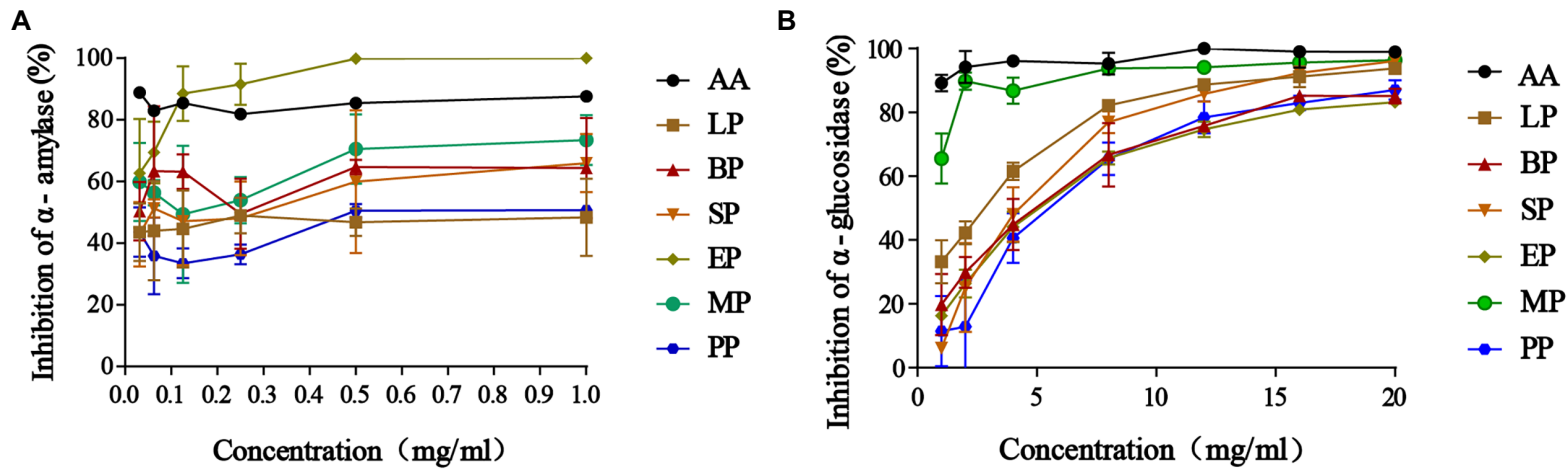
The *α*-amylase inhibitory effects **(A)**; *α*-glucosidase inhibitory effects **(B)**. Data are reported as the mean value ± standard deviation of three replicates.

The inhibitory activity of *α*-amylase was improved in fermented WG groups compared to the control PP group (Figure 2A). The most potent effect was observed at the highest dose, while the lower concentrations of 0.015625–0.25 mg/ml revealed minimum inhibition in SP, MP, LP and BP groups. Furtherly, *α*-amylase inhibition was most prominent in the EP group compared to the SP, MP, LP and BP groups but all inhibition activity was dose-dependent. Moreover, we found that *E. cristatum* fermented WG showed increased *α*-amylase inhibition compared to acarbose at lower concentrations of 0.125 mg/ml, with the inhibition rate of *α*-amylase reaching 99.86% at high concentrations (0.5–1 mg/ml). We further determined the effects on *α*-glucosidase inhibition (Figure 2B) and found that *α*-glucosidase inhibition increased in WG samples in a dose-dependent manner. Similarly, the *α*-glucosidase inhibitory activity of PP, EP and BP groups were from 19.76 to 82.15% at the range of 1–20 mg/ml with no significant differences. Interestingly, the inhibition effect of WG on *α*-glucosidase was enhanced by fermentation with *L. plantarum*, *S cerevisiae* and multiple strains.

### Overview of metabolomics analysis

**Figure 3:**
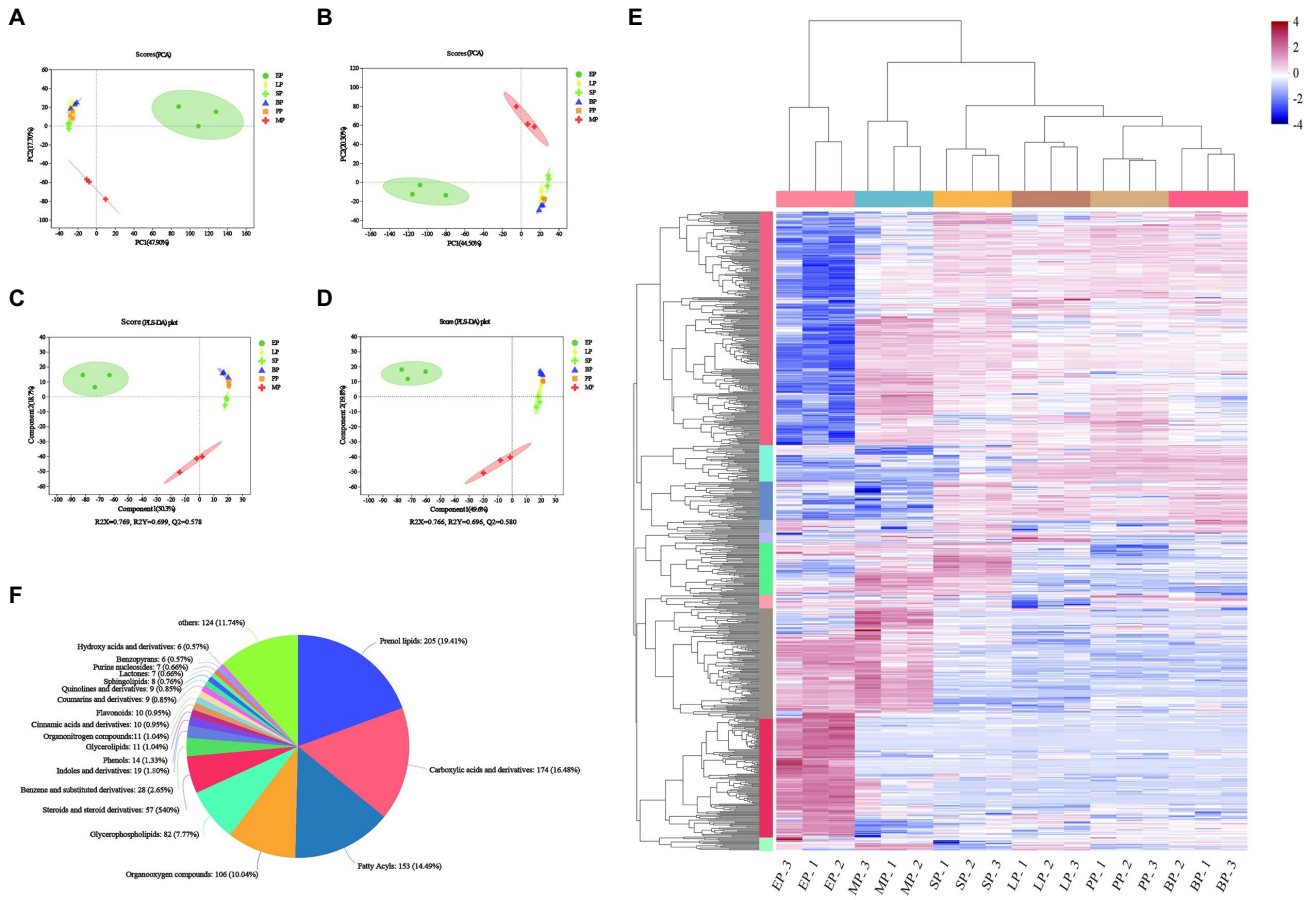
Principal component analysis (PCA) for the significantly differentially expressed metabolites **(A,B)**. Partial least squares discriminant analysis (PLS-DA) for the significantly differentially expressed metabolites **(C,D)**. Clustering analysis of WG sample **(E)**; Classification of all of the detected metabolites. The columns represent 11 groups of WG and each row indicates the same metabolite. The color-coded scale grading from red to blue represents the relative content of metabolites from high to low, respectively **(F)**.

We also used a UHPLC triple TOF high-resolution system, based untargeted metabolomics, to reveal changes in the chemical components of WG fermented by different probiotics in positive and negative ion mode. Ion features with a relative standard deviation greater than 30% were excluded, a total 1,472 compounds were identified (Supplementary Table 1). Subsequently, 817 and 655 variables in the positive and negative modes of the mass spectra were imported to the software for PCA, respectively. In the score plot, 65.6 and 64.8% of the statistical values in the metabolite data were explained by the first two dimensions in the modes of positive and negative ionization, respectively (Figures 3A,B). During fermentation with different strains, LP, SP, BP and PP metabolites were concentrated in one quadrant, while the MP and EP groups were distributed in different quadrants under the positive and negative ion mode, which indicated that the microbial fermentation could effectively alter the metabolic characteristics of WG. PLS-DA was further performed to investigate the metabolite profiles of fermented and unfermented WG, and the data were shown in Figures 3C,D. The results showed that Q2 value was greater than 0.5, and the difference between R2Y and Q2Y was no more than 0.2 in the positive ionization mode, as well as negative ionization mode, indicating good quality of our models. The PLS-DA models showed little discrimination in the metabolic fingerprint of the fermentation in SP, BP and LP groups, but a clear separation was observed in the PLS-DA score plots between EP and MP groups. These indicated that the PLS-DA model was reliable and could be applied to further identify differential metabolites between groups.

To further reveal the dynamic alterations in the metabolites of different groups, a cluster analysis of metabolites was carried out (Figure 3E). The results showed that the overall metabolite content in EP group significantly changed compared to the PP control group, but not in the LP, SP, MP and BP groups. A total of 1,056 compounds were identified and annotated in the Human Metabolome Database compound classification (Figure 3F; Supplementary Table 2). These compouds could be categorized into 21 different classes, including prenol lipids (205, 19.41%); carboxylic acids and derivatives (174, 16.48%); fatty acyls (153, 14.49%); organooxygen compounds (106, 10.04%); glycerophospholipids (82, 7.77%); steroids and steroid derivatives (57, 5.4%); benzene and substituted derivatives (28, 2.65%); indoles and derivatives (19, 1.8%); phenols (14, 1.33%); glycerolipids (11, 1.04%); organonitrogen compounds (11, 1.04%); cinnamic acids and derivatives(10, 0.95%); flavonoids (10, 0.95%); coumarins and derivatives (9, 0.85%); quinolines and derivatives (9, 0.85%); sphingolipids (8, 0.76%); lactones (7, 0.66%); purine nucleosides (7, 0.66%); benzopyrans (6, 0.57%); hydroxy acids and derivatives (6, 0.57%) and others (124, 11.74%).

### Analysis of differential metabolite

**Figure 4:**
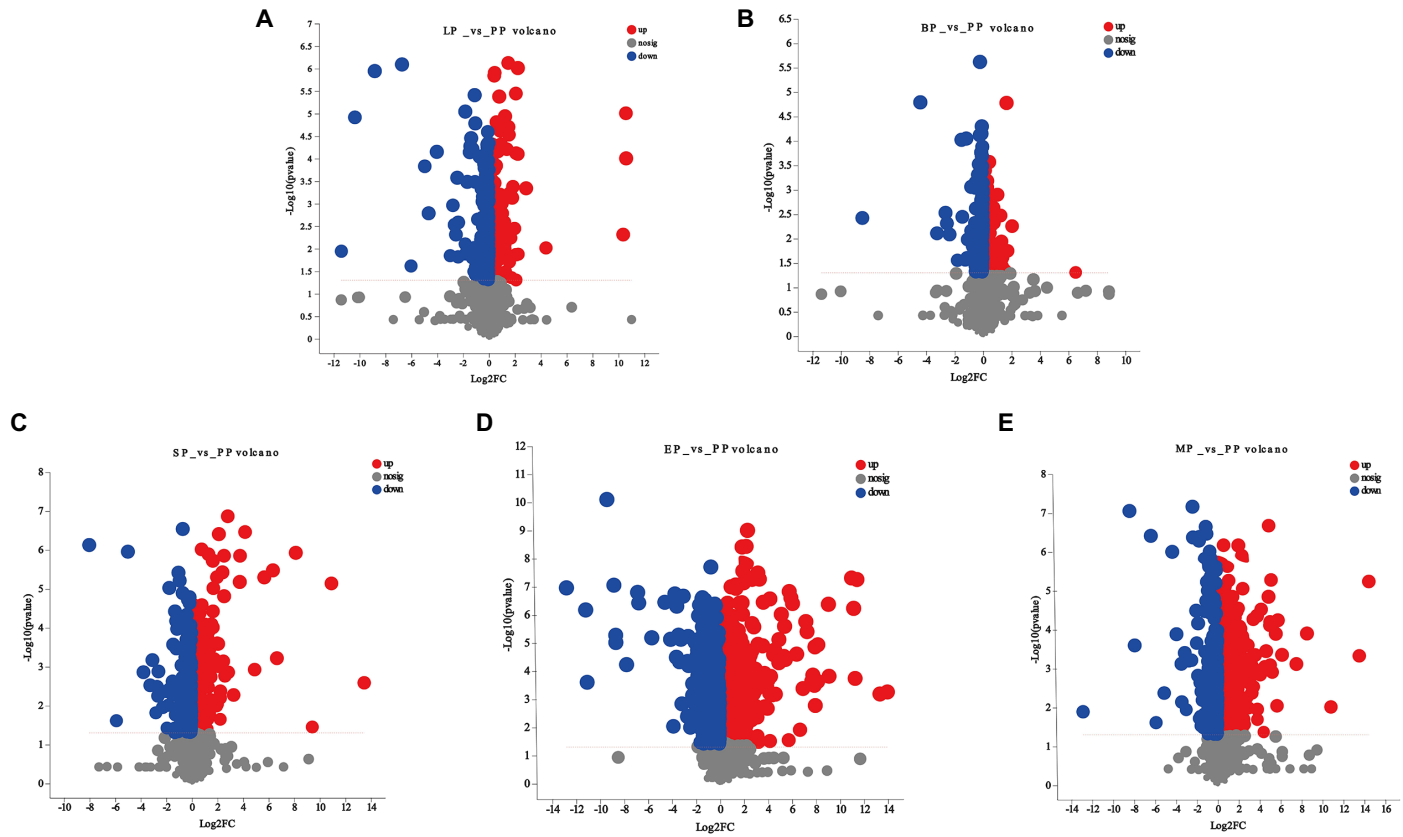
Volcano plot for significant differentially expressed metabolites of **(A)** LP vs. PP; **(B)** BP vs. PP; **(C)** SP vs. PP; **(D)** EP vs. PP; **(E)** MP vs. PP.

One of the aims of this work was to identify the effect of WG metabolites by different probiotics fermentation. Based on the PLS-DA model, differentially expressed metabolites were selected between pairwise comparisons of the five groups according to the following three conditions: VIP value ≥1, *p <* 0.05, and fold-change ≥2 or ≤ 0.5. As shown in Figures 4A–E and Supplementary Table 3, compared to the unfermented group, the largest number of significant metabolites were selected in *E. cristatum* fermented WG with a total of 372 metabolites, including 235 upregulated and 137 downregulated metabolites (EP vs. PP). *L. plantarum* fermentation resulted in 66 differentially expressed metabolites, with a similar number of upregulated and downregulated metabolites (LP vs. PP). *B. licheniformis* fermentation only resulted in 27 differentially expressed metabolites, of which 14 were upregulated and 13 were downregulated (BP vs. PP). *S. cerevisiae* fermentation resulted in 96 differentially expressed metabolites, of which 65 metabolites were upregulated and 31 were downregulated (SP vs. PP). Multi-strain mixed fermentation resulted in 178 metabolites being significantly changed (MP vs. PP) with the number of downregulated metabolites (137) being greater than the number of upregulated metabolites (41). We observed that prenol lipids, carboxylic acids and derivatives, flavonoids, polyphenols, coumarins and its derivatives were the main metabolites in the different fermentation groups, which displayed an important role in the medicinal efficacy of WG.

#### Prenol lipids

In this work, we focused on the the changes in different ginsenosides during fermentation by four probiotics. As shown in Supplementary Table 4, 16 and 40 saponins were detected in the positive and negative ion mode, respectively. We observed that *L. plantarum, S. cerevisiae*, *B. licheniformis* and multi-strain mixed fermentation had little effect on saponins transformation, whereas *E. cristatum* exhibited a stronger biotransformation effect. Additionally, 34 saponins in EP group were downregulated, and 5 were upregulated, which indicated that most saponins in WG fermented by *E. cristatum* showed a decreasing trend, such as Ginsenoside Ra1, Vinaginsenoside R16, Vinaginsenoside R3, Ginsenoside C, Ginsenoside Re, Ginsenoside Rg5, Ginsenoside F2, Ginsenoside B2, Vinaginsenoside R17, Ginsenoside Rh3, Tragopogonsaponin B, Ginsenoside Mc, Cynarasaponin C and Ginsenoside Rf. Interestingly, in the negative ion mode, Cynarasaponin F (C_41_H_64_O_14_, RT = 3.6793, m/z = 801.4078) was significantly increased by 6.1-fold (*p* < 0.05), while Cynarasaponin C (C_42_H_66_O_14_, RT = 3.7301, m/z = 815.4217) was significantly downregulated by 12.5-fold. We speculated that Cynarasaponin C may be converted into Cynarasaponin F by glycosidase secreted by *E. cristatum*. Unfortunately, their pharmacological activity is rarely reported.

#### Carboxylic acids and derivatives

Data obtained in this investigation showed that carboxylic acid and its derivatives accounted for a large proportion of metabolites, including glycerophosphocholines, amino acid, peptides and its analogues, lineolic acids and its derivatives, fatty acids and their conjugates. In the classification of carboxylic acids and derivatives, a total of 30 amino acids, peptides and analogues were screened. Serylhistidine, N-acetyl-L-glutamic acid, Alanyl-Lysine and Seryllysine increased by 539.46-, 198.63-, 65.32- and 18.39-fold in the EP group, respectively. Glutathione has strong antioxidant activity, and we found that its contents were significantly upregulated in EP, SP, MP groups by 3.6-, 1.6- and 4.26-fold, respectively. Similarly, the content of S-(1,2-dicarboxyethyl)glutathione, a derivative of glutathione, increased in all fermentation groups, which indicated that microbial fermentation also displayed an important role on the conversion of carboxylic acids and derivatives in WG.

#### Flavonoids and polyphenols

A total of 13 flavonoids were detected ain the positive and negative ion mode of MS mass spectrometry. *L. plantarum, S. cerevisiae, B. licheniformis, E. cristatum* and mixed strains fermentation had different effects on the content of flavonoids in WG. (S)-Pinocembrin, (−)-Naringenin and Beta-D-Glucosyloxydestruxin B contents were increased in LP and SP groups. The compound 3-Hydroxyflavone content was increased in SP and MP. However, the compound Alpha-Rhamnorobin content was decreased in LP, SP, MP and BP groups. Compared with other strains, the fermentation of *E. cristatum* has the greatest influence on the changes of flavonoids metabolites in WG (3 flavonoids were downregulated and 10 were upregulated). Chrysoeriol (C_16_H_12_O_6_, RT = 4.5783, m/z = 301.0699) is a natural dietary methoxyl flavonoid with an increase of 3.01 times in EP group. Pelargonidin (C_16_H_12_O_6_, RT = 5.6131, m/z = 269.0456) is a natural compound widely found in fruits, which exerts antioxidant, anti-atherosclerosis, anti-inflammatory, anti-hyperglycemia and anti-diabetes effects, increased by 2.45 times in EP group (Lee and Bae, 2019; Guo et al., 2020).

In addition, we compared the differential phenolic metabolites in EP and PP groups, which demonstrated that most monophenolic metabolites appeared a downward trend due to the fermentation of *E. cristatum*, including Vanillin, Cardanoldiene, 6-Gingerol, 2,5-Dimethoxy-4-(2-propenyl)phenol, Sinapyl alcohol, Normetanephrine and Acetaminophen. However, Polyphenols metabolite showed the opposite trend, such as Pyrocatechol (increased by 3.71 times in EP group), which indicated that some monophenols might be converted into polyphenolic compounds by the secreted enzymes of *E. cristatum* during fermentation.

#### Coumarin and its derivatives

A total of 11 coumarins and derivatives were identified in both positive and negative ion mode. Compared with unfermented WG (PP group), the number of differential metabolites detected in LP, BP, SP, MP and EP was 1, 2, 3, 4, 5, respectively. This result indicated the coumarin metabolites in WG were the most affected by the fermentation of *E. cristatum*. In EP group, 8 compounds were upregulated, including Gravelliferone, (R)-Marmin, Trans-grandmarin, Exo-dehydrochalepin, Byssochlamic acid, Xanthotoxol arabinoside and Aesculetin. Among them, (R)-Marmin, Gravelliferone and Byssochlamic acid were significantly increased by 119.55, 9.77 and 6.22 times, respectively. The upregulation of these components could improve the biological activity of WG.

### Correlation between biological activity and metabolites

**Figure 5:**
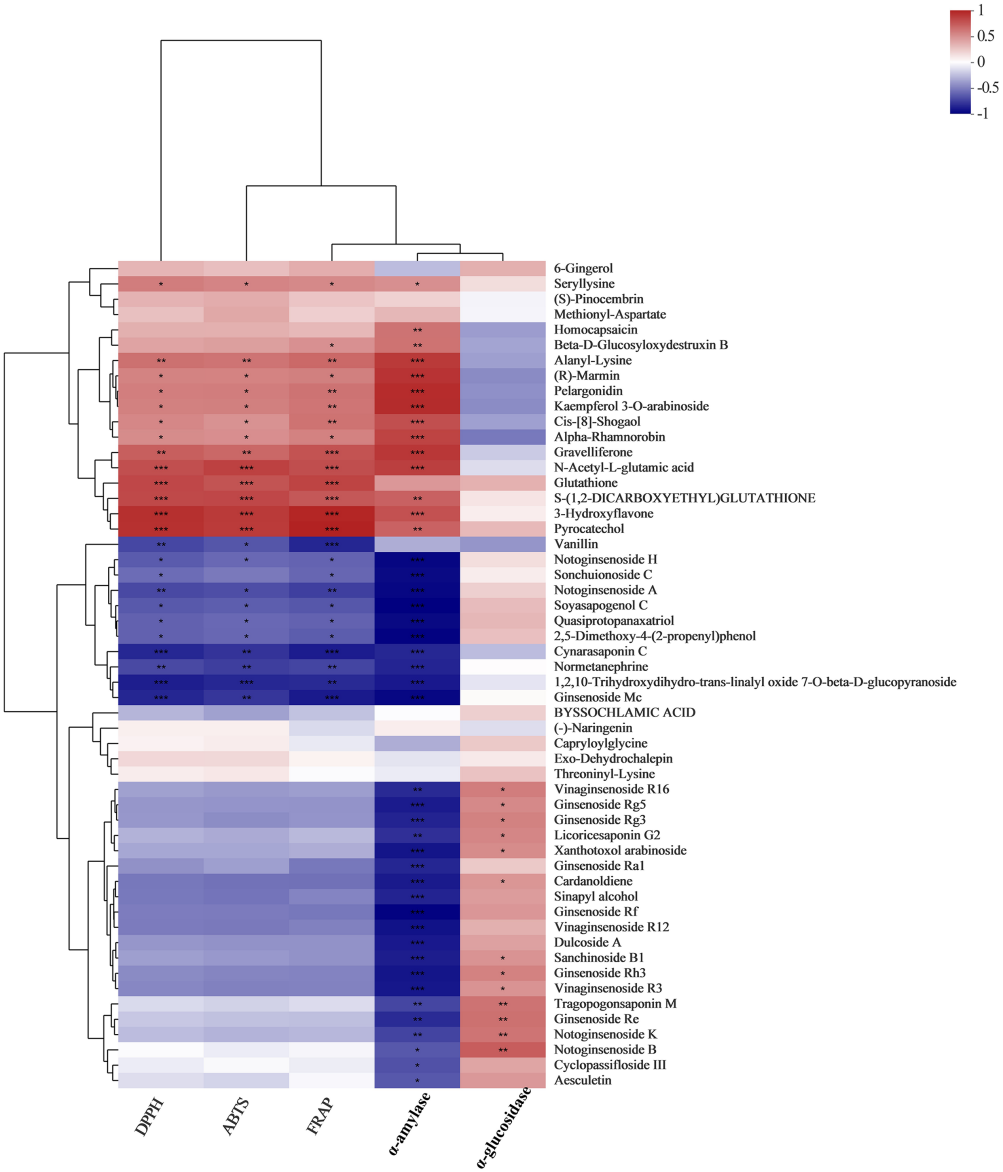
A correlation analysis between fermented WG metabolites and bioactivities was conducted to determine metabolites that potentially contribute to bioactivity.

Correlation coefficients between metabolites and biological activities (DPPH, ABTS, FRAP, *α*-amylase inhibition and *α*-glucosidase inhibition) were shown with heat maps (Figure 5; Supplementary Table 5). Our results indicated that there was a strong correlation between the bioactivities of WG extracts and their metabolite compositions. Some metabolites, such as 6-Gingerol, Methionyl-Aspartate, Seryllysine, (S)-Pinocembrin, Methionyl-Aspartate, Homocapsaicin, Beta-D-Glucosyloxydestru Xin B, Alanyl-Lysine, (R)-Marmin, Pelargonidin, Kaempferol 3-O-arabinoside, Cis-[8]-shogaol, Alpha-Rhamnorobin, Gravelliferone, N-acetyl-L-glutamic acid, Glutathione, S-(1,2-dicarboxyethyl)glutathione, 3-Hydroxyflavone and Pyrocatechol exhibited positive correlations with the DPPH radical scavenging rate, ABTS radical scavenging rate, FRAP reducing power and *α*-amylase inhibition rate. However, saponins and monophenolic metabolites like Vanillin, Notoginsenoside H, Sonchuionoside C, Notoginsenoside A, Soyasapogenol C, Quasiprotopanaxatriol, and 2,5-Dimethoxy-4-(2-propenyl) phenol exhibited strong negative correlations with DPPH radical scavenging rate, ABTS radical scavenging rate and FRAP reducing power. Interestingly, some of saponins, including Vinaginsenoside R16, Ginsenoside Rg5, Ginsenoside Rg3, Licoricesaponin G2, Xanthotoxol arabinoside, Ginsenoside Ra1, Ginsenoside Rf, Vinaginsenoside R12, Sanchinoside B1, Ginsenoside Rh3, Vinaginsenoside R3, Ginsenoside Re, Notoginsenoside K, Notoginsenoside B, Cyclopassifloside III and Aesculetin were negatively correlated with *α*-amylase inhibition, whereas these compounds were positively correlated with *α*-glucosidase inhibition.

## Discussion

Many chronic diseases, including type 2 diabetes, are related to oxidative stress caused by excessive free radicals in the body. In addition, *α*-amylase and *α*-glucosidase can breakdown carbohydrates in food into absorbable monosaccharides *in vivo*, causing high blood glucose levels. Therefore, natural inhibitors of *α*-amylase and *α*-glucosidase from Chinese herbal medicine are excellent potential treatments for diabetes (Oboh et al., 2015; Ríos et al., 2015; Riyaphan et al., 2018; Obafemi et al., 2019). Microbial fermentation is an ideal method for improving the antioxidant and hypoglycemic effects of Chinese herbal medicine. In this work, compared with unfermented WG, the whole fermentation groups, including *L. plantarum*, *B. licheniformis*, *S. cerevisiae*, *E. cristatum* and multi-strain fermentation increased the antioxidant activity and hypoglycemic effects of WG in different degrees. Similar findings were consistent with our results. *S. cerevisiae* fermented BG had increased DPPH radical scavenging activity than unfermented WG (Jung et al., 2017). Microbial fermentation significantly increased the inhibitory activities of *α*-glucosidase and *α*-amylase in blueberry juice (Zhong et al., 2021). However, few studies that improve the inhabitation of *α*-amylase and *α*-glucosidase in Chinese herbal medicine by microbial fermentation were reported. Thus, we believe that microbial fermentation is an excellent method to develop WG related products.

Flavonoids and phenolics, are important secondary metabolites in Chinese herbal medicine, exhibit great antioxidant activity through scavenging free radical (Mosca et al., 2018; Fraga et al., 2019; Destani et al., 2020). In this work, we found that the content of flavonoids and phenolics in WG fermented by four probiotics were significantly different, and *E. cristatum* exhibited the best performance. Most phenolics and flavonoids exist in insoluble bound forms that are covalently bound to the cell-wall structure components. During solid-state fermentation, microbes could secrete abundant hydrolytic enzymes (e.g., amylases, cellulase, *β*-glucosidase and protease) to catalyze the hydrolysis of covalent bonds between cell-wall structure components and insoluble bound phenolics or flavonoids, thereby releasing phenolics and flavonoids (Bei et al., 2017; Chen et al., 2020; Xiao et al., 2021). Herein, we believed that the different hydrolases secreted by the four strains resulted in the difference in content and composition of flavonoids and phenolics in WG.

UPLC-TOF-MS was performed to reveal the effect of probiotics fermentation on metabolite composition in WG. Compared with unfermented group, we identified significant differential metabolites in fermented WG by *L. plantarum*, *B. licheniformis*, *S. cerevisiae* and multiple strains, including purine nucleotides, amino acids, peptides and analogues. These metabolites are mainly involved in the growth and metabolism of the microbial strains. Interestingly, these compounds showed a significant positive correlation with ABTS radical scavenging rate, DPPH radical scavenging rate, ferric reducing antioxidant power, and *α*-amylase inhibitory activity, which indicated that these changed metabolites are responsible for improving the antioxidant and hypoglycemic effects of WG. However, *L. plantarum*, *B. licheniformis*, *S. cerevisiae* and multiple strains fermentation had little effect on WG original chemical composition. Different fermentation methods may influence the conversion of compounds of culture substrates. Previous research reported that RG fermented liquid by *L. plantarum*, the ginsenoside Rg3, Rh2 and Rh1 content increased while ginsenoside Rg2, Rf, Re and Rb1 decreased (Kim et al., 2010). In addition, *Lentilactobacillus buchneri* URN103L was found to hydrolyze major ginsenoside Rb1 into minor ginsenosides Rd. and Rg3 by liquid fermentation (Jung et al., 2019). In this work, we speculated that *L. plantarum*, *B. licheniformis* and *S. cerevisiae* had less of a propagation on solid WG substrates, and the growth of *E. cristatum* was inhibited by other strains in multiple fermentation. All these factors resulted in insufficient secreted enzymes to completely transform the composition of WG. Probably, these strains were more suitable for fermentation under liquid state to obtain complete biotransformation.

Compared to other strains, the solid-state fermentation of *E. cristatum* showed stronger modification and transformation to WG components, due to its strong adapt ability, which allowed the mycelia to grow rapidly on WG substance. At the same time, rich enzymes (i.e., *β*-glucosidase, *α*-amylase, protease, pectinase and cellulase) and various active metabolites can be secreted by *E. cristatum* (Xiao et al., 2021). Some metabolites of WG were significantly changed by fermentation of *E. cristatum*, such as Pelargonidin, Homocapsaicin, Cis-[8]-shogaol, Pyrocatechol, Glutathione, S-(1,2-dicarboxyethyl)glutathione, 3-Hydroxyflavone and (R)-Marmin, which exhibited positive correlations with antioxidant capacity and hypoglycemic effects of WG. However, most saponins showed a downward trend after fermentation by *E. cristatum* in WG, such as Ginsenoside Rh3, Ginsenoside Mc, Ginsenoside Rf, Ginsenoside Ra1, Ginsenoside C, Ginsenoside Re, Ginsenoside Rg5, Ginsenoside F2, Ginsenoside B2 and Ginsenoside Rh3, which showed positive correlations with the inhibition of *α*-amylase, while exhibited strong negative correlations with the inhibition of *α*-glucosidase. Similarly, the solid-state fermented *Panax notoginseng* by *Aspergillus cristatus* reduced the contents of ginsenosides Rb1, Rb2, Rc and Rd. (Lee et al., 2021). Our results showed that the fermentation of *E. cristatum* leaded to the degradation of saponins in WG, and the hypoglycemic effect can be improved by changing the saponin contents.

As a dominant fungus in the fermentation process, the quantity of *E. cristatum* is important criteria for evaluating the quality of Fu brick tea (Xiao et al., 2022). *E. cristatum* contains abundant secondary metabolites, such as emodin methyl ether, chrysophanol and emodin, etc., which had significant inhibitory effects on the *α*-glucosidase activity (Li et al., 2022). Cristatumin F, one of the major bioactive components in *E. cristatum*, showed the modest radical scavenging activity against DPPH radicals (Zou et al., 2014). These findings indicated the potential of *E. cristatum* for enhancement of health effect of WG. In present study, *E. cristatum* fermentation increased the total flavonoid content, the phenolic content, the antioxidant activity and hypoglycemic effects of WG. These results further confirmed that *E. cristatum* was a probiotic strain suitable for fermentation and transformation of Chinese herbal medicine.

In conclusion, we proposed that microbial fermentation was an effective method to improve the biological activity of WG. Furthermore, our data provide new insights into the pharmacological effects and processing of WG.

## Data availability statement

The original contributions presented in the study are included in the article/Supplementary material, further inquiries can be directed to the corresponding author.

## Author contributions

CZ: conceptualization, supervision, revision, validation, and investigation. YC: experiment, software, and writing– original draft preparation. YW, AZ, XZ, LZ, and JZ: resources. YC, YW, and AZ: data curation and project administration. YC and YW: data analysis. CZ and YW: visualization. All authors have read and agreed to the published version of the manuscript.

## Funding

This study was supported by the National Natural Science Foundation of China (Nos. 31860438 and 81760688).

## Conflict of interest

The authors declare that the research was conducted in the absence of any commercial or financial relationships that could be construed as a potential conflict of interest.

## Publisher’s note

All claims expressed in this article are solely those of the authors and do not necessarily represent those of their affiliated organizations, or those of the publisher, the editors and the reviewers. Any product that may be evaluated in this article, or claim that may be made by its manufacturer, is not guaranteed or endorsed by the publisher.

## References

[ref1] Ainsworth E. A. Gillespie K. M. (2007). Estimation of total phenolic content and other oxidation substrates in plant tissues using Folin-Ciocalteu reagent. Nat. Protoc. 2, 875–877. doi: 10.1038/nprot.2007.102, PMID: 17446889

[ref2] An T. Chen M. Zu Z. Chen Q. Lu H. Yue P. (2021). Untargeted and targeted metabolomics reveal changes in the chemical constituents of instant dark tea during liquid-state fermentation by Eurotium cristatum. Food Res. Int. 148, 110623–110623. doi: 10.1016/j.foodres.2021.110623, PMID: 34507767

[ref3] Behera S. S. Ray R. C. Zdolec N. (2018). Lactobacillus plantarum with functional properties: An approach to increase safety and shelf-life of fermented foods. Biomed. Res. Int. 2018:9361614. doi: 10.1155/2018/9361614, PMID: 29998137 PMC5994577

[ref4] Bei Q. Chen G. Lu F. Wu S. Wu Z. (2017). Enzymatic action mechanism of phenolic mobilization in oats (Avena sativa L.) during solid-state fermentation with Monascus anka. Food Chem. 245, 297–304. doi: 10.1016/j.foodchem.2017.10.086, PMID: 29287375

[ref5] Benzie I. Strain J. J. A. B. (1996). The ferric reducing ability of plasma (FRAP) as a measure of "antioxidant power": the FRAP assay. Anal. Biochem. 239, 70–76. doi: 10.1006/abio.1996.0292, PMID: 8660627

[ref6] Chen Y. Wang Y. Chen J. Tang H. Wang C. Li Z. (2020). Bioprocessing of soybeans (Glycine max L.) by solid-state fermentation with Eurotium cristatum YL-1 improves total phenolic content, isoflavone aglycones, and antioxidant activity. RSC Adv. 10, 16928–16941. doi: 10.1039/c9ra10344a, PMID: 35496929 PMC9053166

[ref7] Chen Y. C. Yu Y. H. (2022). Bacillus licheniformis-fermented products and enramycin differentially modulate microbiota and antibiotic resistome in the cecal digesta of broilers. Poult. Sci. 101:102010. doi: 10.1016/j.psj.2022.102010, PMID: 35841645 PMC9293667

[ref8] Dai C. Hou Y. Xu H. Huang L. Dabbour M. Mintah B. K. (2022). Effect of solid-state fermentation by three different bacillus species on composition and proteinstructure of soybean meal. J. Sci. Food Agric. 102, 557–566. doi: 10.1002/jsfa.11384, PMID: 34145902

[ref9] Destani F. Naccarato A. Tagarelli A. Cassano A. (2020). Recovery of Aromatics from Orange juice evaporator condensate streams by reverse osmosis. Membranes 10:92. doi: 10.3390/membranes10050092, PMID: 32397308 PMC7281282

[ref10] Di Cagno R. Filannino P. Vincentini O. Cantatore V. Cavoski I. Gobbetti M. (2019). Fermented Portulaca oleracea L. juice: a novel functional beverage with potential ameliorating effects on the intestinal inflammation and epithelial injury. Nutrients 11:248. doi: 10.3390/nu11020248, PMID: 30678049 PMC6412393

[ref11] Dong L. Li Y. Xu J. Yang J. Wei G. Shen L. (2019). Biofertilizers regulate the soil microbial community and enhance Panax ginseng yields. Chin. Med. 14, 20–14. doi: 10.1186/s13020-019-0241-1, PMID: 31143242 PMC6533694

[ref12] Eldarov M. A. Kishkovskaia S. A. Tanaschuk T. N. Mardanov A. V. (2016). Genomics and biochemistry of Saccharomyces cerevisiae wine yeast strains. Biochemistry 81, 1650–1668. doi: 10.1134/S0006297916130046, PMID: 28260488

[ref13] Fahy E. Subramaniam S. Brown H. A. Glass C. K. Merrill A. H. Murphy R. C. (2005). A comprehensive classification system for lipids. J. Lipid Res. 46, 839–861. doi: 10.1194/jlr.E400004-JLR200 15722563

[ref14] Fraga C. G. Croft K. D. Kennedy D. O. Tomás-Barberán F. A. (2019). The effects of polyphenols and other bioactives on human health. Food Funct. 10, 514–528. doi: 10.1039/c8fo01997e, PMID: 30746536

[ref15] Guo L. Kang J. S. Kang N. J. Je B. I. Lee Y. J. Park Y. H. (2020). Pelargonidin suppresses adipogenesis in 3T3-L1 cells through inhibition of PPAR-γ signaling pathway. Arch. Biochem. Biophys. 686:108365. doi: 10.1016/j.abb.2020.108365, PMID: 32315651

[ref16] He M. Huang X. Liu S. Guo C. Xie Y. Meijer A. H. (2018). The difference between White and red ginseng: variations in Ginsenosides and immunomodulation. Planta Med. 84, 845–854. doi: 10.1055/a-0641-6240, PMID: 29925101

[ref17] Jin D. Zhang Y. Zhang Y. Duan L. Zhou R. Duan Y. (2021). Panax Ginseng C.a.Mey. As medicine: the potential use of Panax Ginseng C.a.Mey. As a remedy for kidney protection from a pharmacological perspective. Front. Pharmacol. 12:734151. doi: 10.3389/fphar.2021.734151, PMID: 34512359 PMC8426624

[ref18] Jung K. An J. M. Eom D. W. Kang K. S. Kim S. N. (2017). Preventive effect of fermented black ginseng against cisplatin-induced nephrotoxicity in rats. J. Ginseng Res. 41, 188–194. doi: 10.1016/j.jgr.2016.03.001, PMID: 28413323 PMC5386130

[ref19] Jung J. Jang H. J. Eom S. J. Choi N. S. Lee N. K. Paik H. D. (2019). Fermentation of red ginseng extract by the probiotic lactobacillus plantarum KCCM 11613P: ginsenoside conversion and antioxidant effects. J. Ginseng Res. 43, 20–26. doi: 10.1016/j.jgr.2017.07.004, PMID: 30662290 PMC6323145

[ref20] Kim B. G. Choi S. Y. Kim M. R. Suh H. J. Park H. J. (2010). Changes of ginsenosides in Korean red ginseng (Panax ginseng) fermented by lactobacillus plantarum M1. Process Biochem. 45, 1319–1324. doi: 10.1016/j.procbio.2010.04.026

[ref21] Kim J. K. Cui C. H. Yoon M. H. Kim S. C. Im W. T. (2012). Bioconversion of major ginsenosides Rg1 to minor ginsenoside F1 using novel recombinant ginsenoside hydrolyzing glycosidase cloned from Sanguibacter keddieii and enzyme characterization. J. Biotechnol. 161, 294–301. doi: 10.1016/j.jbiotec.2012.06.021, PMID: 22766417

[ref22] Lee I. C. Bae J. S. (2019). Pelargonidin protects against renal injury in a mouse model of sepsis. J. Med. Food 22, 57–61. doi: 10.1089/jmf.2018.4230, PMID: 30160593

[ref23] Lee J. W. Ji S. H. Choi B. R. Choi D. J. Lee Y. G. Kim H. G. (2018). UPLC-QTOF/MS-based metabolomics applied for the quality evaluation of four processed Panax ginseng products. Molecules 23, 2062–2062. doi: 10.3390/molecules23082062, PMID: 30126124 PMC6222836

[ref24] Lee S. Reddy C. K. Ryu J. J. Kyung S. Lim Y. Park M. S. (2021). Solid-state fermentation with Aspergillus cristatus enhances the Protopanaxadiol- and Protopanaxatriol-associated skin anti-aging activity of Panax notoginseng. Front. Microbiol. 12:602135. doi: 10.3389/fmicb.2021.602135, PMID: 34975775 PMC8718098

[ref25] Li P. Zhu X. Xiao M. Su Y. Yu S. Tang J. (2022). Rapid isolation and hypoglycemic activity of secondary metabolites of Eurotium cristatum by high-speed countercurrent chromatography. J. Chromatogr. Sci. doi: 10.1093/chromsci/bmac020, PMID: [Epub ahead of print]. 35325046

[ref26] Liu N. Song M. Wang N. Wang Y. Wang R. An X. (2020). The effects of solid-state fermentation on the content, composition and in vitro antioxidant activity of flavonoids from dandelion. PLoS One 15, e0239076–e0239076. doi: 10.1371/journal.pone.0239076, PMID: 32931505 PMC7491732

[ref27] Managa M. G. Akinola S. A. Remize F. Garcia C. Sivakumar D. (2021). Physicochemical parameters and bioaccessibility of lactic acid bacteria fermented chayote leaf (Sechiumedule) and pineapple (Ananas comosus) smoothies. Front. Nutr. 8:649189. doi: 10.3389/fnut.2021.649189, PMID: 33898502 PMC8058202

[ref28] Mosca F. Hidalgo G. I. Villasante J. Almajano M. P. (2018). Continuous or batch solid-liquid extraction of antioxidant compounds from seeds of Sterculia apetala plant and kinetic release study. Molecules 23:1759. doi: 10.3390/molecules23071759, PMID: 30021965 PMC6100467

[ref29] Obafemi T. O. Olaleye M. T. Akinmoladun A. C. (2019). Antidiabetic property of miracle fruit plant (Synsepalum dulcificum Shumach. & Thonn. Daniell) leaf extracts in fructose-fed streptozotocin-injected rats via anti-inflammatory activity and inhibition of carbohydrate metabolizing enzymes. J. Ethnopharmacol. 244, 112124–112124. doi: 10.1016/j.jep.2019.112124, PMID: 31374224

[ref30] Oboh G. Akinbola I. A. Ademosun A. O. Sanni D. M. Odubanjo O. V. Olasehinde T. A. (2015). Essential oil from clove bud (Eugenia aromatica Kuntze) inhibit key enzymes relevant to the Management of Type-2 diabetes and some pro-oxidant induced lipid peroxidation in rats pancreas in vitro. J. Oleo Sci. 64, 775–782. doi: 10.5650/jos.ess14274, PMID: 25994557

[ref31] Pang J. Zhu X. Liu Y. Fu J. Zhao X. Yang M. (2016). Spectral analysis of Chinese medicinal herbs based on delayed luminescence. Evid. Based Complement. Alternat. Med. 2016, 8469024–8469028. doi: 10.1155/2016/8469024, PMID: 27478482 PMC4958485

[ref32] Prasad B. J. Sharavanan P. S. Sivaraj R. (2019). Efficiency of Oryza punctata extract on glucose regulation: inhibition of α-amylase and α-glucosidase activities. Grain Oil Sci. Technol. 2, 44–48. doi: 10.1016/j.gaost.2019.04.007

[ref33] Purnomo Y. Makdasari J. Fatahillah F. I. (2021). Inhibitory activity of Urena lobata leaf extract on alpha-amylase and alpha-glucosidase: in vitro and in silico approach. J. Basic Clin. Physiol. Pharmacol. 32, 889–894. doi: 10.1515/jbcpp-2020-0430, PMID: 34214371

[ref34] Renchinkhand G. Magsar U. Bae H. C. Choi S. H. Nam M. S. (2022). Identification of β-Glucosidase activity of Lentilactobacillus buchneri URN103L and its potential to convert Ginsenoside Rb1 from Panax ginseng. Foods 11:529. doi: 10.3390/foods11040529, PMID: 35206006 PMC8870947

[ref35] Ríos J. L. Francini F. Schinella G. R. (2015). Natural products for the treatment of type 2 diabetes mellitus. Planta Med. 81, 975–994. doi: 10.1055/s-0035-1546131 26132858

[ref36] Riyaphan J. Jhong C. H. Lin S. R. Chang C. H. Tsai M. J. Lee D. N. (2018). Hypoglycemic efficacy of docking selected natural compounds against α-Glucosidase and α-amylase. Molecules 23:2260. doi: 10.3390/molecules23092260, PMID: 30189596 PMC6225388

[ref37] Shi M. Wei Y. Nie Y. Wang C. Sun F. Jiang W. (2020). Alterations and correlations in microbial community and Metabolome characteristics in generalized aggressive periodontitis. Front. Microbiol. 11:573196. doi: 10.3389/fmicb.2020.573196, PMID: 33329431 PMC7734087

[ref38] Shibata S. (2001). Chemistry and cancer preventing activities of ginseng saponins and some related triterpenoid compounds. J. Korean Med. Sci. 16, S28–S37. doi: 10.3346/jkms.2001.16.S.S28, PMID: 11748374 PMC3202208

[ref39] Syed Salleh S. N. A. Mohd Hanapiah N. A. Ahmad H. Wan Johari W. L. Osman N. H. Mamat M. R. (2021). Determination of Total Phenolics, flavonoids, and antioxidant activity and GC-MS analysis of Malaysian stingless bee Propolis water extracts. Scientifica (Cairo) 2021, 3789351–3789351, 3789311. doi: 10.1155/2021/3789351, PMID: 34721923 PMC8556095

[ref40] Wang C. Liu J. Deng J. Wang J. Weng W. Chu H. (2020). Advances in the chemistry, pharmacological diversity, and metabolism of 20(R)-ginseng saponins. J. Ginseng Res. 44, 14–23. doi: 10.1016/j.jgr.2019.01.005, PMID: 32095093 PMC7033361

[ref41] Wang L. Zhang H. Lei H. (2021). Phenolics profile, antioxidant activity and flavor volatiles of pear juice: influence of lactic acid fermentation using three lactobacillus strains in monoculture and binary mixture. Foods 11, 11–11. doi: 10.3390/foods11010011, PMID: 35010138 PMC8750113

[ref42] Wu W. Sun L. Zhang Z. Guo Y. Liu S. (2015). Profiling and multivariate statistical analysis of Panax ginseng based on ultra-high-performance liquid chromatography coupled with quadrupole-time-of-flight mass spectrometry. J. Pharm. Biomed. Anal. 107, 141–150. doi: 10.1016/j.jpba.2014.12.030, PMID: 25590943

[ref43] Wu X. Yao H. Cao X. Liu Q. Cao L. Mu D. (2017). Production of vinegar from purple sweet potato in a liquid fermentation process and investigation of its antioxidant activity. 3 Biotech. 7:308. doi: 10.1007/s13205-017-0939-7, PMID: 28955605 PMC5595722

[ref44] Xiao Y. He C. Chen Y. Ho C. T. Wu X. Huang Y. (2022). UPLC-QQQ-MS/MS-based widely targeted metabolomic analysis reveals the effect of solid-state fermentation with Eurotium cristatum on the dynamic changes in the metabolite profile of dark tea. Food Chem. 378:131999. doi: 10.1016/j.foodchem.2021.131999, PMID: 35081481

[ref45] Xiao Y. Wu X. Yao X. Chen Y. Ho C. T. He C. (2021). Metabolite profiling, antioxidant and α-glucosidase inhibitory activities of buckwheat processed by solid-state fermentation with Eurotium cristatum YL-1. Food Res. Int. 143, 110262–110262. doi: 10.1016/j.foodres.2021.110262, PMID: 33992363

[ref46] Yoon D. Shin W. C. Lee Y. S. Kim S. Baek N. I. Lee D. Y. (2020). A comparative study on processed Panax ginseng products using HR-MAS NMR-based metabolomics. Molecules 25:1390. doi: 10.3390/molecules25061390, PMID: 32197517 PMC7146337

[ref47] Yu S. Zhou X. Li F. Xu C. Zheng F. Li J. (2017). Microbial transformation of ginsenoside Rb1, re and Rg1 and its contribution to the improved anti-inflammatory activity of ginseng. Sci. Rep. 7, 138–110. doi: 10.1038/s41598-017-00262-0, PMID: 28273939 PMC5428039

[ref48] Zhang H. M. Li S. L. Zhang H. Wang Y. Zhao Z. L. Chen S. L. (2012). Holistic quality evaluation of commercial white and red ginseng using a UPLC-QTOF-MS/MS-based metabolomics approach. J. Pharm. Biomed. Anal. 62, 258–273. doi: 10.1016/j.jpba.2012.01.010, PMID: 22310552

[ref49] Zhang J. Ma Z. Zheng L. Zhai G. Wang L. Jia M. (2014). Purification and antioxidant activities of intracellular zinc polysaccharides from Pleurotus cornucopiae SS-03. Carbohydr. Polym. 111, 947–954. doi: 10.1016/j.carbpol.2014.04.074, PMID: 25037435

[ref50] Zhong H. Abdullah Z. M. Tang J. Deng L. Feng F. (2021). Probiotics-fermented blueberry juices as potential antidiabetic product: antioxidant, antimicrobial and antidiabetic potentials. J. Sci. Food Agric. 101, 4420–4427. doi: 10.1002/jsfa.11083, PMID: 33421121

[ref51] Zhou S. S. Auyeung K. K. Yip K. M. Ye R. Zhao Z. Z. Mao Q. (2020). Stronger antiobesity effect of white ginseng over red ginseng and the potential mechanisms involving chemically structural/compositional specificity to gut microbiota. Phytomedicine 74:152761. doi: 10.1016/j.phymed.2018.11.021, PMID: 31005370

[ref52] Zou M. Zhang W. Dong Q. Tang C. Cao F. Su E. (2021). Submerged fermentation of Ginkgo biloba seed powder using Eurotium cristatum for the development of ginkgo seeds fermented products. J. Sci. Food Agric. 101, 1782–1791. doi: 10.1002/jsfa.10792, PMID: 32892346

[ref001] Zou X. Li Y. Zhang X. Li Q. Liu X. Huang Y. (2014). A new prenylated indole diketopiperazine alkaloid from Eurotium cristatum. Molecules 19:17839–17847. doi: 10.3390/molecules191117839 25372398 PMC6271712

